# Supplementary material for: Association between oral microbiome and seven types of cancers in East Asian population: a two-sample Mendelian randomization analysis
Source: Front Mol Biosci. 2023 Nov 21;10:1327893. doi: 10.3389/fmolb.2023.1327893 (PMC10702768; doi:10.3389/fmolb.2023.1327893)
Supplement: Supplementary file 1 [file DataSheet1.ZIP › Supplementary Materials/MR plots for tongue/tongue═╝/Pancreatic cancer/╥╚╧┘░⌐_╔╕╤í╜ß╣√/pheno.1388_to_pancreatic cancer_leave_one_out.pdf]

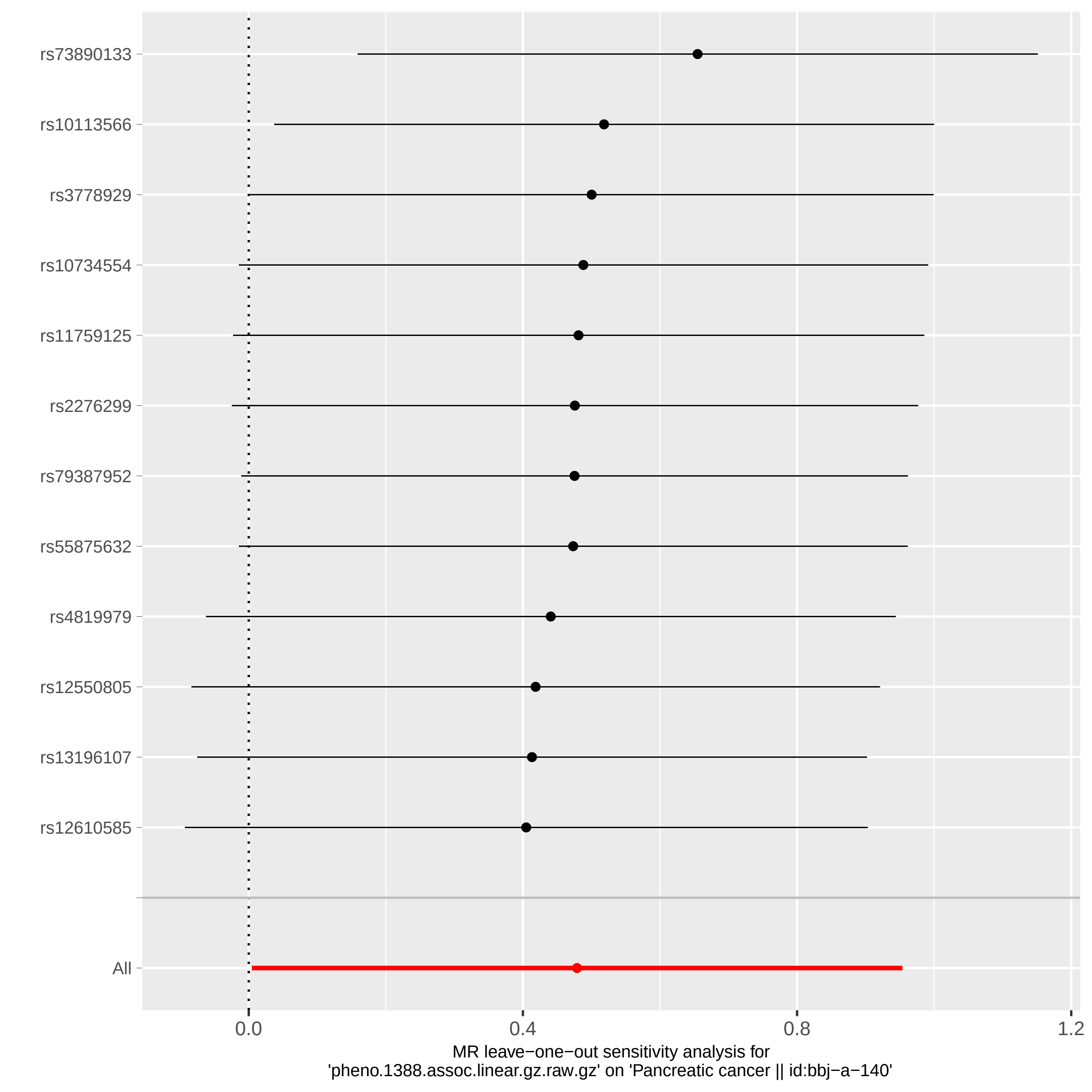
rs73890133
rs10113566
rs3778929
rs10734554
rs11759125
rs2276299
rs79387952
rs55875632
rs4819979
rs12550805
rs13196107
rs12610585
All
0.0
0.4
0.8
1.2
MR leave−one−out sensitivity analysis for
'pheno.1388.assoc.linear.gz.raw.gz' on 'Pancreatic cancer || id:bbj−a−140'
